# A synthetic data strategy for Scotland: Using synthetic data to improve access to public sector data for research

**DOI:** 10.23889/ijpds.v8i2.2215

**Published:** 2023-09-14

**Authors:** Lynne Adair

**Affiliations:** 1 Research Data Scotland, Edinburgh, United Kingdom

## Objectives

Research Data Scotland (RDS) is working to improve the economic, social and environmental wellbeing in Scotland by enabling access to, and linkage of, public-sector data for research in the public good. Our objective was to explore how synthetic data can be used to support this aim.

## Method

We investigated what other, similar, data organisations were doing around synthetic data, both in Scotland and beyond, and the issues to consider. We discussed use cases, tools, level of fidelity of synthesis, disclosure risk, information governance (IG) requirements, where the synthetic data might sit, who could access it, training and accreditation requirements, and barriers to synthesis. The findings were used to draft a synthetic data strategy, create a working group and plan future work. We also held a user workshop to discuss researcher requirements and asked our public panel about their understanding and concerns around synthetic data.

## Results

Three workstreams have been set up around disclosure risk & IG, synthesis, and access, promotion & engagement. A test synthesis of education data is planned. From our user workshop one of the main themes that emerged was the usefulness of low fidelity, unlinked datasets for data discovery purposes at the early stages of a project. Other areas where synthetic data can improve data access are for training, where synthetic data can be used to upskill users in the use of administrative data, and in code development, to allow earlier access to data for code generation and to reduce the time needed in secure settings and with access to real data. Future work will bring together data controllers to discuss disclosure risk.

## Conclusion

Synthetic data can potentially be used to improve and speed up data access. RDS will fund and support synthetic data projects to demonstrate value, and work with data controllers to produce synthetic datasets on an ongoing basis, for public sector research datasets in Scotland.

